# Detecting SARS-CoV-2 cryptic lineages using publicly available whole genome wastewater sequencing data

**DOI:** 10.1371/journal.ppat.1012850

**Published:** 2025-06-09

**Authors:** Reinier Suarez, Devon A. Gregory, David A. Baker, Clayton A. Rushford, Torin L. Hunter, Nicholas R. Minor, Clayton M. Russ, Emma E. Copen, David H. O’Connor, Marc C. Johnson

**Affiliations:** 1 Department of Molecular Microbiology and Immunology, University of Missouri-School of Medicine, Columbia, Missouri, United States of America; 2 Department of Pathology and Laboratory Medicine, University of Wisconsin-Madison, Madison, Wisconsin, United States of America; Fred Hutchinson Cancer Center, UNITED STATES OF AMERICA

## Abstract

Beginning in early 2021, unique and highly divergent lineages of SARS-CoV-2 were sporadically found in wastewater sewersheds using a sequencing strategy focused on **amplifying** the most **rapidly evolving** region of SARS-CoV-2, the receptor binding domain (RBD). Because these RBD sequences did not match known circulating strains and their source was not known, we termed them “cryptic lineages”. To date, more than 20 cryptic lineages have been identified using the RBD-focused sequencing strategy. Here, we identified and characterized additional cryptic lineages from SARS-CoV-2 wastewater sequences submitted to NCBI’s Sequence Read Archives (SRA). Wastewater sequence datasets were screened for individual sequence reads that contained combinations of mutations frequently found in cryptic lineages but not contemporary circulating lineages. Using this method, we identified 18 cryptic lineages that appeared in multiple (2–81) samples from the same sewershed, including 12 that were not previously reported. Partial consensus sequences were generated for each cryptic lineage by extracting and mapping sequences containing cryptic-specific mutations. Surprisingly, seven of the mutations that appeared convergently in cryptic lineages were reversions to sequences that were highly conserved in SARS-CoV-2-related enteric bat Sarbecoviruses. The apparent reversion to bat Sarbecovirus sequences is consistent with the notion that SARS-CoV-2 adaptation to replicate efficiently in respiratory tissues preceded the COVID-19 pandemic.

## Author summary

Wastewater surveillance has been used during the SARS-CoV-2 pandemic to monitor viral activity and the spread of viral lineages. Occasionally, SARS-CoV-2 sequences from wastewater reveal unique evolutionary advanced lineages of SARS-CoV-2 from an unknown source, which are termed cryptic lineages. Many groups nationwide also use wastewater surveillance to track the virus and upload that information to NCBI’s SRA database. That sequence data was screened to identify 18 cryptic lineages worldwide and identify convergent mutations throughout the genome of cryptic lineages that suggest reversion to residues common in SARS-CoV-2-related Sarbecoviruses.

## Introduction

Wastewater surveillance has been widely used to identify chemicals and microbes [1–3]. During the SARS-CoV-2 pandemic, this technique gained prominence for its efficient tracking of various variants of concern [4]. Our group began tracking SARS-CoV-2 lineages from wastewater in early 2021, and in March 2021, we discovered the first instance of an evolutionarily advanced SARS-CoV-2 receptor binding domain (RBD) haplotype that appeared repeatedly in a single sewershed, which we later termed a “cryptic lineage” [5]. Examples of cryptic lineages have now been reported worldwide [5–11]. Similarities between genomes from persistent SARS-CoV-2 infections in immunocompromised patients and cryptic lineages suggest these may reside within immunocompromised individuals [8,12,13]. Furthermore, a single cryptic lineage derived from a lineage that stopped circulating in early 2021 was traced to a commercial building in late 2022, and 12S ribosomal RNA sequencing of the wastewater indicated that the only meaningful species contributing to the wastewater was human [13]. Therefore, cryptic lineages are believed to be derived from individuals with very long SARS-CoV-2 infections.

Cryptic lineages often forecast mutations that are eventually acquired by circulating lineages. For instance, Spike substitutions N440K, S477N, E484A, and Y505H had not been seen in any major circulating lineages prior to Omicron. Yet, these mutations had repeatedly appeared in cryptic lineages long before Omicron emerged [5,6]. Cryptic lineage mutations have also forecast numerous changes that were acquired by later Omicron lineages [13]. The convergence between mutations found in cryptic lineages and those eventually found in circulating lineages suggests that cryptic lineages and major circulating lineages share some selective pressures. However, many of the mutations seen repeatedly in cryptic lineages have yet to become prominent in any major circulating lineage [13]. It is unknown whether major circulating lineages will eventually acquire those mutations or whether those mutations account for selective pressures that differ from circulating lineages.

Around the world, many organizations use whole genome sequencing (WGS) to detect and identify SARS-CoV-2 variants in wastewater samples. Much of this data is uploaded to the National Center for Biotechnology Information’s (NCBI) Sequencing Read Archive (SRA) or one of its international equivalents, the International Nucleotide Sequence Database Collection (INSDC). In this report, we screen 135,672 samples from over 2000 sites across 45 countries and demonstrate the feasibility of screening the SRA database to detect SARS-CoV-2 cryptic lineages and analyze their mutations.

## Results

Using conservative thresholds, our lab has identified over 20 cryptic lineages by amplifying the RBD sequence from SARS-CoV-2 RNA in wastewater samples [5,6,13]. From the previously discovered cryptic lineages, we compiled a list of mutations observed in cryptic lineages that were not commonly found in circulating lineages (S1 Fig). This list of 69 amino acid substitutions in the spike RBD was termed “cryptic lineage-defining amino acid substitutions.”

Using the search terms “SARS-CoV-2 wastewater”, we downloaded wastewater SARS-CoV-2 sequence reads from SRA that were available on February 18th, 2024 (S2 Data), that had sample collection dates on or before October 31 2023, mapped these reads to the SARS-CoV-2 genome (NC_045512), and processed them with the program SAM Refiner [14]. We identified individual sequencing reads in the SRA datasets that contained at least two of the cryptic lineage-defining amino acid substitutions (S1 Data). These were analyzed using automated scripts followed by manual validation to identify haplotypes that did not match any known sequence from a patient sample and appeared in at least two samples from the same sewershed. Using the subset of identified sequences, we found sequencing reads consistent with 18 independent cryptic lineages. Of the 18 identified lineages, three of the lineages we reported previously and three of the lineages had been reported by other groups [5–7,9,11,13]. The duration of detection varied widely among the cryptic lineages; the shortest time a cryptic lineage was detected was one month (CA-1 and NY-2), while two cryptic lineages were detected for over a year (UK-1 and WI-1) (Table 1).

**Table 1:** List of cryptic lineages identified along with their location, sample size, and dates they were detected.

# of Samples	Cryptic Location	ID	Coverage	Nextclade Derived	Usher Derived	Cryptic Lineages Detected	Parent Circulated
3	California	CA-1	47.56%	AY.103	B.1.617.2	September 2023	May 2021 – January 2022
6	Switzerland	CH-1	44.41%	B.1.416.1		February – July 2021	January 2020 – August 2020
5	Colorado	CO-1	11.43%	AY.4		December 2021 – March 2022	January 2020 – August 2020
2	Florida	FL-1	20.13%	B.1.533		July 2022	May 2020 – June 2021
2	Florida	FL-2	25.44%	AY.35		December 2022 – January 2023	June 2021 – August 2021
8	Kentucky	KY-1	38.75%	AY.3		February – June 2022	June 2021 – December 2021
5	Michigan	MI-1	73.97%	B.1.1.7	B.1.1.7	September 2022 – May 2023	September 2020 – September 2021
20	Netherlands	NL-1	59.33%	AY.43	AY.43	August – September 2022	June 2021 – August 2021
15	New York	NY-1	63.47%	B.1.2	B.1.2	October 2021 – February 2022	May 2020 – June 2021
2	New York	NY-2	37.39%	B.1.336		July 2021	January 2020 – August 2020
4	New York	NY-3	35.96%	B.1.503		June – August 2021	April 2020 – August 2020
3	New York	NY-4	22.83%	D.2		June – August 2023	December 2020 – January 2021
6	New York	NY-5	35.58%	B.1.623		May – July 2023	January 2021 – May 2021
4	New York	NY-6	24.17%	B.1.1.7		May – June 2023	September 2020 – September 2021
4	New York	NY-7	21.46%	R.1		July – September 2023	December 2020 – June 2021
38	Ohio	OH-1	44.38%	B	B.1	July 2022 – June 2023	January 2020 – October 2021
48	United Kingdom	UK-1	50.28%	B.1.566	B.1	November 2020 – February 2022	January 2020 – October 2021
81	Wisconsin	WI-1	31.72%	B.1.234		April 2022 – August 2023	June 2020 – April 2021

Coverage indicates the fraction of the genome that could be unambiguously assigned to the cryptic lineage through linkage to a cryptic specific mutation. The phylogenetic software programs Nextclade [15] and Usher [16] were used to determine which variant the cryptic lineages originated from. The time periods when the cryptic lineages were detected and the time periods when the cryptic lineage parent lineages were circulating are indicated.

**Fig 1:**
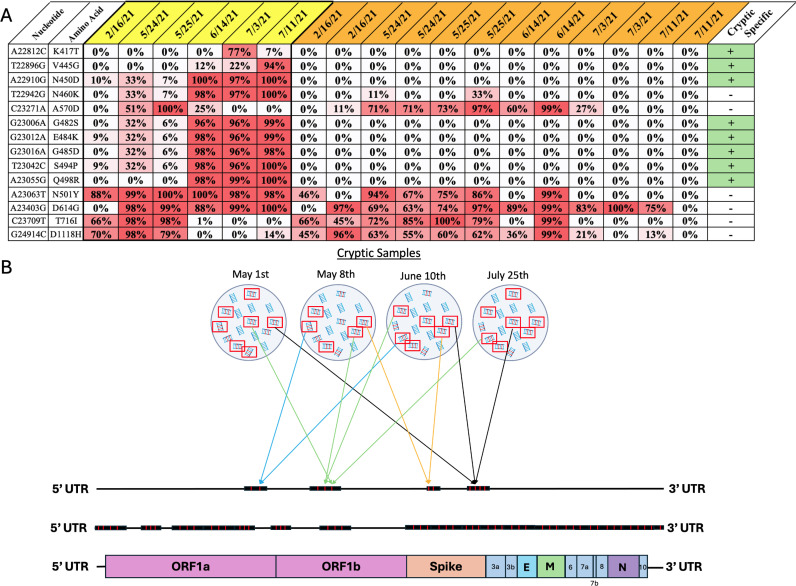
Schematic of workflow. Samples from sewer shed facilities containing cryptic lineages (yellow) were compared against samples from neighboring sewer sheds that did not contain cryptic lineages (orange). **A)** Using the CH-1 cryptic lineage as an example, mutations found in at least two cryptic samples, with a prevalence of 50x more in the cryptic samples, are tentatively considered cryptic-specific (green). **B)** The sequence reads containing cryptic-specific mutations (red box) were mapped onto the SARS-CoV-2 genome, with varying coverage across the genome to create a consensus sequence (middle genome). To be mapped onto the genome, a cryptic-specific sequence must appear in two or more samples.

**Fig 2:**
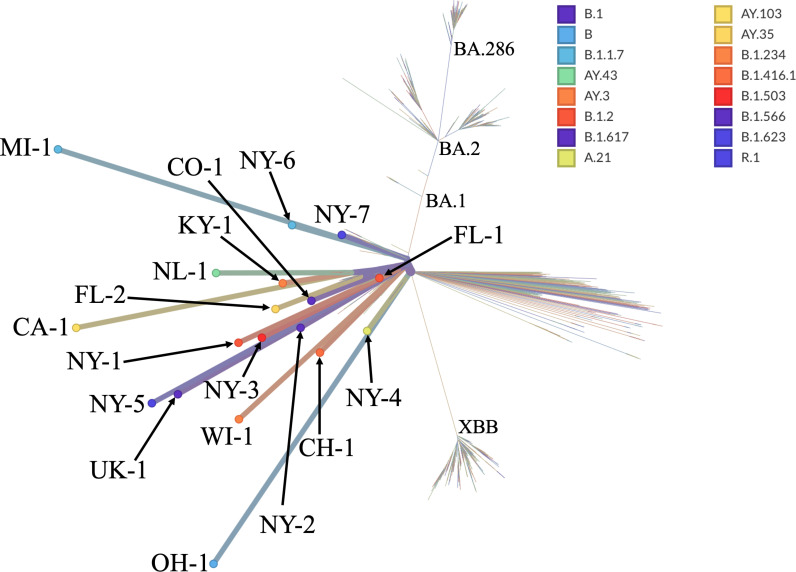
The phylogenetic tree generated by NextClade illustrates the diversity of the cryptic lineages. The consensus sequences were uploaded onto Nextclade and compared against the Wuhan-Hu-1/2019 (MN908947). The phylogenetic tree highlights the diversity among the cryptic lineages detected.

After cryptic lineages were identified based on their RBD sequence, we retrospectively identified other datasets from the same sewershed that share cryptic-defining characteristics outside of the RBD to partially reconstruct lineage genomes. We compared the individual SARS-CoV-2 sequences present in wastewater samples from sewersheds containing cryptic lineages to the sequences from samples from neighboring (same state) sewersheds collected during the same time period and, when possible, sequenced by the same agency (S2 and S3 Data). Individual mutations that appeared in at least two samples from the cryptic sewershed and were at least 50x more prevalent in the cryptic-containing sewershed than in neighboring sewersheds were considered putatively cryptic-specific mutations (Fig 1a). Additionally, any mutation frequently appearing in the same sequence read as the cryptic-specific mutation was presumed to be present in the cryptic lineage (Fig 1b; see methods for specific criteria). This process was repeated with all 18 cryptic lineages to approximate the polymorphisms present in each lineage (S4 and S5 Data). A consensus sequence was generated for each cryptic lineage using its cryptic-specific mutations and sequences that appeared on the same read as the cryptic-specific mutations (Fig 1b, S6 Data). Generating a complete consensus sequence for each cryptic lineage was not possible in most cases because regions were excluded that did not have any cryptic-defining mutations. As a result, sequence coverage varied between cryptic lineages, with the highest coverage being 73.97% (MI-1) and the lowest 11.43% (CO-1). The consensus sequence was used as input for the phylogenetic software programs UShER [16] and Nextclade [15] to determine its predicted parent SARS-CoV-2 lineage (Table 1). All the cryptic lineages were predicted to be derived from lineages that stopped circulating months to years prior to their detection in wastewater (Table 1). A phylogenetic tree of the cryptic lineages illustrates the extreme diversity of these lineages (Fig 2). The use of a consensus sequence, which is derived from a mixture of diverse lineages with a shared common ancestor, could potentially influence the branch lengths in the phylogenetic tree, and may not fully capture the true diversity within each cryptic lineage.

**Fig 3:**
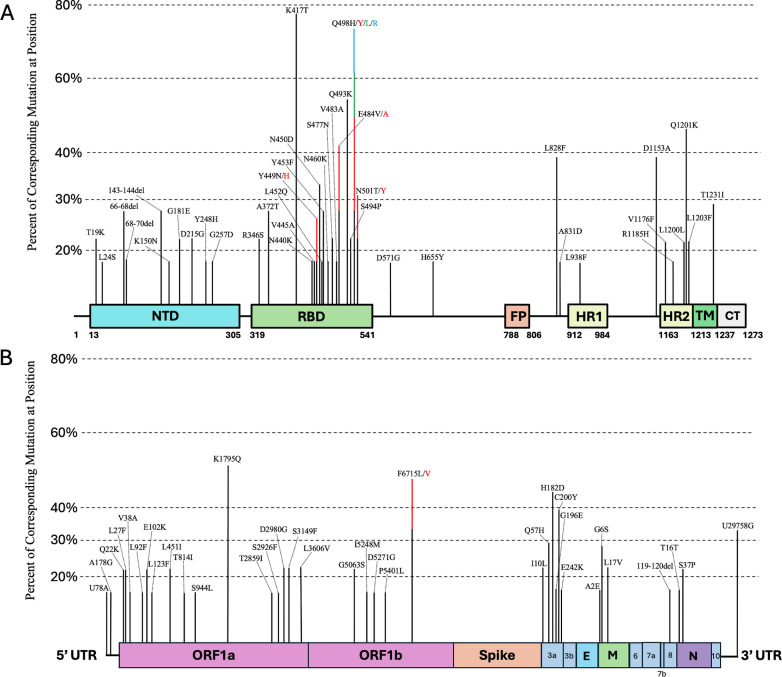
Convergent cryptic changes found in **≥** **3 lineages.** **A)** Convergent mutations that appeared in at least three cryptic lineages were mapped onto the spike protein based on their location and prevalence across all the cryptic lineages. **B)** Convergent non-Spike mutations mapped against the SARS-CoV-2 genome. Positions which contain multiple mutations in the same position are represented as stacked bars and color-coded.

Interestingly, we observed the same mutations in the consensus sequence appearing in multiple independent cryptic lineages. Such convergent changes are unlikely to be sequencing artifacts and likely reflect adaptation to common selective pressures. Mutations that appeared in three or more cryptic lineages were mapped onto a diagram of the SARS-CoV-2 genome while excluding mutations found in the consensus sequence of the parent lineages (Fig 3a, 3b; S7 Data). We observed 85 nucleotide changes in at least three cryptic lineages. The most common changes in Spike were K417T (78%) and Q493K (56%), which have been shown to affect antibody escape [17,18] and may also have small effects on ACE2 binding, with effects ranging from slightly increasing to modestly decreasing binding depending on the genetic background [19]. Although K417T was present in the Gamma variant of concern and a few Omicron sub-lineages, such as BA.2.18, it has been present in less than 1% of circulating lineages found in people. By contrast, Q493K is extremely rare and has not been a lineage-defining change in any named PANGO lineage. The most common cryptic-specific mutations outside the Spike were in ORF1a (K1795Q) and ORF3a (H182D), each observed in 50% of the identified cryptic lineages.

Among the 85 nucleotide changes that occurred convergently in at least three cryptic lineages, 79 changed a protein sequence through non-synonymous changes or deletions. Of the four changes that did not alter a protein sequence, two were silent (C25162A/Spike: L1200L, 22.22%), and three were in non-coding regions (T78A (16.67%), A178G (16.67%), and T29758G (33.33%)). Interestingly, we observed that the Spike change C25162A (L1200L) was always associated with the neighboring C25163A (Q1201K) change. These two mutations together create the sequence TCTAAAAGAACT, which is a near-perfect match to the consensus SARS-CoV-2 transcription regulatory sequence (TRS) TCTAAACGAACT [20]. Although C25162A and C25163A are relatively rare in patient sequences [21], the two changes usually occur together (>60% of the time). While the function of this additional TRS is not known, it is a possible explanation for the convergence of the silent C25162A change.

A particularly notable convergent non-coding change in the cryptic lineages is at the 3’ UTR of the SARS-CoV-2 genome, T29758G. This mutation is in the highly conserved region of the stem-loop two motif (s2m), which is found in many Coronaviruses and other RNA viruses [22–24]. Remarkably, the s2m in SARS-CoV-2 deviates from the consensus s2m found in other RNA viruses, including Sarbecoviruses, and the T29758G mutation restores SARS-CoV-2 to the consensus s2m sequence [24,25]. The s2m stem-loop is not essential for replication as the sequence was deleted in Omicron lineage BA.2 and all of its derivatives; thus, it has been nearly absent in circulating lineages for over two years [26]. However, in the case of the cryptic lineages, the sequence frequently reverts the SARS-CoV-2 s2m to the Sarbecovirus consensus sequence.

Several of the most common convergent changes in cryptic lineages, such as ORF1a: K1795Q and T29758G, were conversions to the sequence found in closely related bat Sarbecoviruses such as RaTG-13. Although SARS-CoV-2 is a human respiratory pathogen, the most closely related Sarbecoviruses primarily infect Horseshoe bats and are primarily believed to be enteric pathogens. To explore if other convergent changes in cryptic lineages represent reversions to the Sarbecovirus consensus sequence, the sequences of seven closely related Sarbecoviruses (RpYN06, RaTG-13, BANAL-52, BANAL-103, BANAL-116, BANAL-236, and BANAL-247) were compared to SARS-CoV-2 to identify amino acid positions that were conserved across all seven sarbecoviruses, but differed in the original SARS-CoV-2 A and B lineages.

**Fig 4:**
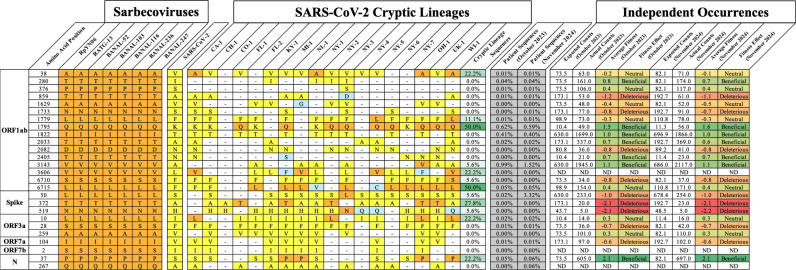
Chart of SARS-CoV-2 amino acids that deviate from the consensus Sarbecovirus amino acid sequence. Consensus amino acids found across seven bat Sarbecoviruses (orange) that are different in SARS-CoV-2 (yellow). The amino acid positions where a change is observed but differ from the Sarbecoviruses and SARS-CoV-2 are highlighted in blue. Frequency of patient sequences, reported by CoV-Spectrum [21], reverting to the Sarbecovirus consensus by October 2023 or November 2024 are shown. The independent occurrence calculated from Bloom and Neher’s calculator [26] for each mutation over the same time periods is shown, including its fitness score and effect. Mutations that did not appear in Bloom and Neher’s calculator were designated as not determined (ND).

A total of 26 amino acid substitutions were identified where the SARS-CoV-2 sequence differed from all seven of the bat sarbecoviruses. Of these 26 positions, 12 substitutions in cryptic lineages had reverted to the Sarbecovirus consensus sequence in at least one cryptic lineage, and seven of the reversions occurred in at least three cryptic lineages (Fig 4). As of October 31st, 2023, in these 26 positions, none had reverted to the Sarbecovirus sequence in over 1% of US patient SARS-CoV-2 sequences. However, low prevalence in patient sequences does not necessarily mean that mutations cannot provide a fitness advantage. A study by Bloom and Neher examined how frequently particular mutations were independently acquired by SARS-CoV-2 relative to how frequently they would be expected to be acquired by random chance [27]. From this, they assigned a fitness score to individual SARS-CoV-2 mutations. Of the seven Sarbecovirus reversions that were acquired by at least three cryptic lineages, only two were predicted to be advantageous. Interestingly, the change predicted to be the most deleterious is Spike: A372T. This position has been noted previously because all Sarbecoviruses except SARS-CoV-2 contain a predicted glycosylation site at position 370, and A372T restores this site. In addition to the five cryptic lineages that contained A372T, two other cryptic lineages also restore this glycosylation site through changes S371N and A372DEL. It has been experimentally demonstrated that restoring glycosylation to SARS-CoV-2 through the A372T mutation reduces viral replication in human lung cells [28]. In addition, it has been demonstrated that abolishing the glycosylation site at position 370 in bat Sarbecoviruses makes the virus highly sensitive to trypsin digestion at pH 5.5 [29]. Thus, the frequent reversion to the consensus bat sarbecovirus sequence in cryptic lineages is consistent with cryptic lineages being subject to some similar selective pressures as that of its enteric progenitors.

**Fig 5:**
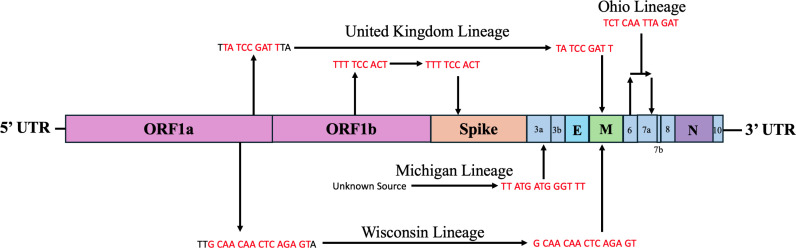
Insertion sequences were mainly derived from duplications. Insertion sites were mapped onto the SARS-CoV-2 genome to visually represent where the duplicated sequence (red) occurred and where the insertion was detected with respect to the cryptic lineage.

Five of the cryptic lineages were found to have small insertions (Fig 5). Three of the insertions occurred in the ectodomain of the structural proteins, specifically in the spike and M genes, as was previously noted for one cryptic lineage, and the other two insertions occurred in non-structural genes, ORF3a and ORF7a [13]. A closer observation of the inserted nucleotide sequence revealed that four of the five insertions were duplicated sequences from other parts of the SARS-CoV-2 genome.

**Fig 6:**
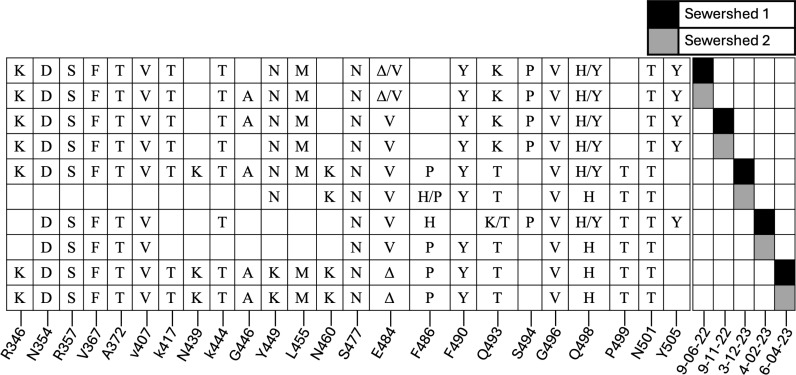
Ohio’s cryptic-specific RBD mutations over time. Both locations shared highly similar mutation profiles in the RBD, with distinct mutations appearing in both locations around the same time (N460K, F486P, and P499T). Crossed-out cells signify areas of low or no coverage.

One cryptic lineage was detected in SRA datasets from two different sewersheds separated by approximately 40 miles. Samples were independently obtained from both sewersheds and tested for the presence of the cryptic lineage. Samples from both sewersheds contained a cryptic lineage that closely matched the sequence observed in the SRA sequences (S2 and S3 Figs). Similar to our finding with the cryptic lineage found in Wisconsin (WI-1) [13], the sequence from the Ohio cryptic lineage did not remain static over a nine-month period (Fig 6). Both sewersheds from Ohio shared highly similar cryptic-specific mutation profiles throughout the dates detected in the SRA. Notably, mutations in the Spike protein, specifically N460K, F486P, Q493T, and P499T, were detected for the first time on the same date from both sewer sheds, strongly suggesting that this lineage was being deposited into wastewater from a single source, likely a person who commuted between the two locations. The Ohio cryptic lineage persisted until June 2023 before disappearing. The successful detection of the Ohio cryptic lineage from independently obtained samples serves as a valuable validation of the method’s ability to detect cryptic lineages from public repositories.

## Discussion

Screening NCBI’s SRA database for cryptic lineages underestimates the prevalence of these lineages. Our screen relies on the detection of specific changes that are common to cryptic lineages, but there may be other cryptic lineages that do not harbor these conserved cryptic lineage signatures. Moreover, only a subset of global wastewater sequences are submitted to SRA, and the cryptic lineages need to be sufficiently abundant that their sequences can be detected after dilution with all of the other material in the sewershed. Finally, our genome reconstruction method assumes that there are not multiple cryptic lineages present in any sewersheds, and the reconstructions would not be accurate if there were in fact multiple cryptic lineages present. Despite these limitations, the method of cryptic lineage detection described here effectively detects cryptic lineages worldwide and highlights cryptic-specific polymorphisms outside the RBD. More importantly, this method illustrated cryptic-specific convergent polymorphisms across the many cryptic lineages.

Five insertion sites occur in various parts of the SARS-CoV-2 genome, but the impacts of these insertions are unknown. The insertions occurring in the structural regions of the SARS-CoV-2 genome (spike and M genes) are in the ectodomain section of the proteins. Studies have shown that SARS-CoV anti-M, in conjunction with anti-Spike, enhances the neutralizing capability of the virus [30–32]. Thus, these insertions may contribute to immunological escape, while the significance of the escape requires testing. The role of the insertions in ORF3a and ORF7a are unknown; however, it is evident that SARS-CoV-2 readily utilizes the strategy of insertions as a form of adaptation to different selection pressures. Furthermore, it should be emphasized that these insertions are not unique to cryptic lineages and have been previously observed in patient samples [33].

The K1795Q substitution is in the papain-like protease domain of nsp3, and the substitution has been shown to enhance the ability of the protease to cleave polyubiquitin chains [34]. The most parsimonious explanation for the reversion of sequences in cryptic lineages to the sequence found in closely related Sarbecoviruses is that cryptic lineages could be subject to selective pressures in common with enteric bat Sarbecoviruses that are not imposed on circulating lineages of SARS-CoV-2 that are primarily respiratory. The observation that enteric viruses consistently appear at >100 times higher levels than respiratory viruses in wastewater suggests that the digestive tract acts as a selective filter, diminishing much of the signal from respiratory viruses. This aligns with the observation that cryptic SARS-CoV-2 lineages, which are detected in wastewater and thought to originate from a single individual, are shed at extraordinarily high levels [13]. Furthermore, the observations of cryptic lineages reverting to sequences found in their enteric ancestors, combined with their extremely high shedding rates, could be consistent with the idea that cryptic SARS-CoV-2 lineages are predominantly replicating in the gastrointestinal (GI) tract.

The observation that SARS-CoV-2 contains at least seven distinct substitutions that convergently changed to the sequence found in enteric Sarbecoviruses suggests a possible selective pressure to maintain the Sarbecovirus consensus sequence at these positions. It is plausible that the observed reversions may be attributed to the inherent fitness advantage conferred by consensus amino acids at these positions. This phenomenon has been well-documented in evolutionary studies of retroviruses, particularly HIV-1, where the consensus sequence often represents an optimal fitness peak within the adaptive landscape [35]. However, the observation that many of the changes have an apparent negative impact on circulating viruses suggests that the fitness advantage may be condition-dependent. The fact that SARS-CoV-2 had changes at each of these positions when it began circulating in humans suggests that SARS-CoV-2 had replicated in a non-enteric environment for a long enough period of time to allow these substitutions to persist and become fixed in the viral genomes that started the COVID-19 pandemic.

## Methods

### NCBI SRA screening

All SARS-CoV-2 sequencing reads were obtained through the NCBI’s SRA and found by using the search terms “SARS-CoV-2 wastewater” then filtered to exclude any sample collected after October 2023. Raw reads were downloaded and mapped to the SARS-CoV-2 genome (NC_045512) using Minimap2 [36] and the resulting sam file processed by SAM Refiner with the parameters ‘—wgs 1—collect 0—indel 0—covar 0—min_count 1—min_samp_abund 0—min_col_abund 0—ntabund 0—ntcover 1’. Unique sequence outputs from SAM Refiner were programmatically screened for a combination of specific amino acid changes only found in cryptic lineages, with positive hits manually examined to exclude false positives such as rare patient lineages or sequences with obviously high error rates. All scripts used in this study are publicly available through Github: https://github.com/dholab/SRA_wastewater_lineages.

### Cryptic-specific polymorphisms

To assess polymorphisms from sequence read runs (SRRs) containing cryptic lineages, we compared the sequences from sewer sheds containing cryptic lineages to sequences from neighboring (sewer sheds from the same state) sewer sheds that did not contain cryptic lineages. Two non-cryptic SRRs (negative samples) were compared against an SRR with a cryptic sequence. We selected negative and positive samples processed by the same sequencing agency to rule out testing bias. The selected SRRs were then processed using SAM Refiner, and the unique_seq and covar outputs were processed by a custom script to determine mutations associated with each cryptic lineage. The parameters for each cryptic-specific mutation are as follows: 1) The mutation must be present in SRA reads from two or more samples from a sewer shed where a cryptic lineage was observed; 2) the average sum abundance for the mutation must be 50x greater in the cryptic sewer sheds than in the non-cryptic sewer sheds; 3) a sum abundance of >10% of the maximum sum abundance of the most abundant polymorphism for a cryptic-specific mutation from those sewer shed samples. To account for mutations prevalent in both circulating and cryptic lineages, any polymorphism appearing at least 75% of the time in the same sequence read as a cryptic-specific polymorphism is considered part of the cryptic lineage and reported as “linked.”

The script generates three files for each cryptic lineage: a “CommonVars” file that lists all the polymorphisms found in all the samples compared (S3 Data), a “Cryptic_CommonVars” file containing all the cryptic-specific mutations while flagging Delta, RaTG13, ubiquitous, and linked mutations (S4 Data), and a “Cryptic_Covar” file that lists all the polymorphisms that were linked to cryptic-specific polymorphisms (S5 Data). The cryptic-specific polymorphisms are then aggregated into a new file using a script that sorts them based on their prominence in all the cryptic lineages, while excluding mutations found in the parent lineages (S7 Data). The cryptic-specific polymorphisms with a prevalence of ≥3 across all the cryptic lineages were then mapped onto a diagram of the SARS-CoV-2 genome based on their respective site.

### Ohio cryptic lineage wastewater sample processing and RNA extraction

24-hour composite samples of wastewater were collected weekly from the inflow of two undisclosed wastewater treatment facilities in Ohio. Samples arrived in 50mL conical tubes and were stored at 4°C until processed. Samples were centrifuged at 3000xg for 10 minutes and filtered through a 0.22μM polyethersulfone membrane (Millipore, Burlington, MA, USA). Approximately 37.5mL of wastewater was mixed with 12.5mL solution containing 50% (w/vol) polyethylene glycol 8000 and 1.2M NaCl, mixed, and incubated at 4°C. The samples would then be spun down at 12,000 RCF for 2 hours at 4°C. The supernatant was decanted, and the RNA was extracted from the remaining pellet using the QIAamp Viral RNA Mini Kit (Qiagen, Germantown, MD, USA) following the manufacturer’s instructions. The RNA was extracted in a final volume of 60uL.

### Amplifying the ohio cryptic lineage

The primary RBD RT-PCR was performed using the Superscript IV One-Step RT-PCR System (ThermoFisher Scientific,12594100, Waltham, MA, USA). Primary RT-PCR amplification was performed as follows: [25°C (2:00) + 50°C (20:00) + 95°C (2:00)] + ([95°C (0:15) + 55°C (0:30) + 72°C (1:00)] × 25) cycles using the MiSeq primary PCR primers 5’-CAAACTTCTAACTTTAGAGTCCAACC-3’ and 5’-AAGTCCACAAACAGTTGCT-3’, and an additional reaction was conducted to exclude Omicron lineages using the primer sets 5’-CCCTGATAAAGAACAGCAACC-3’ and 5’-TATATAATTCCGCATCATTTTCCAC-3’. A secondary nested PCR (25μL) was performed on RBD amplifications using 5μL of the primary PCR as the template with MiSeq nested gene-specific primers containing 5′ adapter sequences (0.5μM each). The MiSeq nested RBD primer set to amplify all the lineage amplicons are 5’-gtgactggagttcagacgtgtgctcttccgatctACTACTACTCTGTATGGTTGGTAAC-3’ and 5’-acactctttccctacacgacgctcttccgatctCCTAATATTACAAACTTGTGCCCTT-3’, while the MiSeq nested RBD primer set to amplify excluded Omicron amplicons are 5’-acactctttccctacacgacgctcttccgatctGTGATGAAGTCAGACAAATCGC-3’ and 5’-gtgactggagttcagacgtgtgctcttccgatctATGTCAAGAATCTCAAGTGTCTG-3’, with the addition of dNTPs (100μM each) (New England Biolabs, N0447L) and Q5 DNA polymerase (New England Biolabs, M0541S, Ipswich, MA, USA). Secondary PCR amplification was performed as follows: 95°C (2:00) + [95°C (0:15) + 55°C (0:30) + 72°C (1:00)] × 20 cycles. A tertiary PCR (50μL) was performed to add adapter sequences required for Illumina cluster generation with forward and reverse primers (0.2μM each), dNTPs (200μM each) (New England Biolabs, N0447L, Ipswich, MA, USA) and Phusion High- Fidelity or (KAPA HiFi for CA samples) DNA Polymerase (1U) (New England Biolabs, M0530L, Ipswich, MA, USA). PCR amplification was performed as follows: 98°C (3:00) + [98°C (0:15) + 50°C (0:30) + 72°C (0:30)] × 7 cycles +72°C (7:00). Amplified product (10μl) from each PCR reaction was combined and thoroughly mixed to make a single pool. Pooled amplicons were purified by adding Axygen AxyPrep MagPCR Clean-up beads (Corning, MAG-PCR-CL-50, Corning, NY, USA) or in a 1.0 ratio to purify final amplicons. The final amplicon library pool was evaluated using the Agilent Fragment Analyzer automated electrophoresis system (Agilent, Santa Clara, CA, USA), quantified using the Qubit HS dsDNA assay (ThermoFisher Scientific, Waltham, MA, USA), and diluted according to Illumina’s standard protocol. The Illumina MiSeq instrument generated paired-end 300 base pair reads (Illumina, San Diego, CA, USA). Adapter sequences were trimmed from output sequences using Cutadapt.

Sequencing reads were processed as previously described [14]. VSEARCH tools merged paired reads and dereplicated sequences [37]. Dereplicated sequences from RBD amplicons were mapped to the reference sequence of SARS-CoV-2 (NC_045512.2) using Minimap2 [35]. Mapped amplicon sequences were then processed with SAM Refiner using the same spike sequence as a reference and the command line parameters “--Alpha 1.8 --foldab 0.6” [14]. The haplotypes representing the Ohio lineages were rendered into figures using plotnine (https://plotnine.org).

### Phylogenetic analysis

Phylogenetic trees were developed utilizing the software programs Nextclade [15] and UShER [16] using their default parameters. Each cryptic lineage had a consensus fasta file generated using the sequence reads containing cryptic-specific mutations (S5 Dataset). Non-cryptic specific mutations, which appeared at least 75% of the time in the same sequence read as a cryptic-specific mutation, are assumed to be part of the cryptic lineage and thus included in the consensus sequence. Positions with no linkage to a cryptic defining mutation were masked by designating ‘N’. This ensures the tree is built only on portions where the sequence was unambiguous. To accurately generate the consensus sequence, only the last 35 positive cryptic lineage samples were used to create the consensus sequence. In Nextclade, consensus sequences were uploaded to the program, and each consensus was compared to the SARS-CoV-2 sequence (Wuhan-Hu-1/2019 (MN908947)). Using UShER, consensus sequences were copied onto the designated field and compared using the phylogenetic tree version “16,472,770 genomes from GISAID, GenBank, COG-UK and CNCB”.

## Supporting information

S1 Fig Compiled list of Spike RBD mutations not found in major circulating lineages but frequently found in cryptic lineages. For an SRA sequence read to be counted as cryptic, it must contain at least two of the mutations listed.
(DOCX)

S2 Fig SARS-CoV-2 haplotype from the second location in Ohio. RBD-focused amplifications of samples collected from the second location in Ohio. Amplifications using the Omicron exclusion primer sets are designated as ALT.
(DOCX)

S3 Fig SARS-CoV-2 haplotype from the first Ohio location. RBD-focused amplifications of samples collected from the first location in Ohio. Amplifications using the Omicron exclusion primer sets are designated as ALT.
(DOCX)

S1 Data Screening of sequence reads that contained a combination of at least two polymorphisms from S1_Figure. This data lists the sequence read, its count and abundance, the SRR ID associated with the sequence, and the location from which it was sampled.
(XLSX)

S2 Data A list of all the samples (SRRs) screened in this study, along with their location and date sampled. (XLSX)

S3 Data Referred to as “CommonVars”, this data file contains a list of all the polymorphisms found in both the cryptic positive SRRs (yellow) and cryptic negative SRRs (orange). This data highlights the count and abundance of each polymorphism in each sample and its sum in the positive, negative, and across all samples. To comply with submission guidelines, many low-abundance polymorphisms were excluded from the analysis. A comprehensive report is available upon request from the corresponding author.
(XLSX)

S4 Data Referred to as “Cryptic_CommonVars”, this data file contains all the cryptic-specific polymorphisms found in the CommonVars data file. Additionally, this data highlights the count and abundance of each cryptic-specific polymorphism, in addition to flagging polymorphisms that appeared in the same sequence read as a cryptic-specific polymorphism (linked) and polymorphisms found abundantly in both positive and negative samples (ubiquitous). Additionally, this file flagged polymorphisms associated with the Delta SARS-CoV-2 lineage and the bat coronavirus, RATG13. Yellow-highlighted SRRs indicate positive samples, while orange-highlighted SRRs indicate negative samples.
(XLSX)

S5 Data Referred to as “Cryptic_Covar”, this data file contains all linked polymorphisms. This data file highlights the count and abundance of linked polymorphisms and the SRRs in which they are found.
(XLSX)

S6 Data List all fasta assemblies for all SARS-CoV-2 cryptic lineages identified. Regions or positions that displayed low or no coverage were designated as “N”.
(DOCX)

S7 Data This data file contains a compilation of all cryptic-specific polymorphisms across all cryptic lineages. (XLSX)
