# Supplementary material for: Detecting SARS-CoV-2 cryptic lineages using publicly available whole genome wastewater sequencing data
Source: PLoS Pathog. 2025 Jun 9;21(6):e1012850. doi: 10.1371/journal.ppat.1012850 (PMC12176291; doi:10.1371/journal.ppat.1012850)
Supplement: S1 Fig — For an SRA sequence read to be counted as cryptic, it must contain at least two of the mutations listed. (DOCX) [file ppat.1012850.s001.docx]

| List of Cryptic Spike RBD Mutations |
| --- |
| G413R G413K K417T K417R D420N N439K N440E N440D N440H N440R L441R K444DEL K444S V445DEL V445DEL V445G V445R V445N V446DEL G446T G446N G446D G446V G447C N447DEL Y448DEL Y449N Y449H Y449R Y449S Y453F R454K L455M L455W F456V N460S T470N I472L S477D V483DEL V483A V483I E484DEL E484P E484Q E484V E484D E484T F486H F486A F490H F490Y F490V Q493K G496V Q498H Q498Y Q498K P499S P499T P499H T500S N501S N501T 445A 450k 478Q 493T 493V 498L 504D T478R N450D K444N F456L L455F A475V F486P S494P |
