# Supplementary material for: Detecting SARS-CoV-2 cryptic lineages using publicly available whole genome wastewater sequencing data
Source: PLoS Pathog. 2025 Jun 9;21(6):e1012850. doi: 10.1371/journal.ppat.1012850 (PMC12176291; doi:10.1371/journal.ppat.1012850)
Supplement: S3 Fig — RBD-focused amplifications of samples collected from the first location in Ohio. Amplifications using the Omicron exclusion primer sets are designated as ALT. (DOCX) [file ppat.1012850.s003.docx]

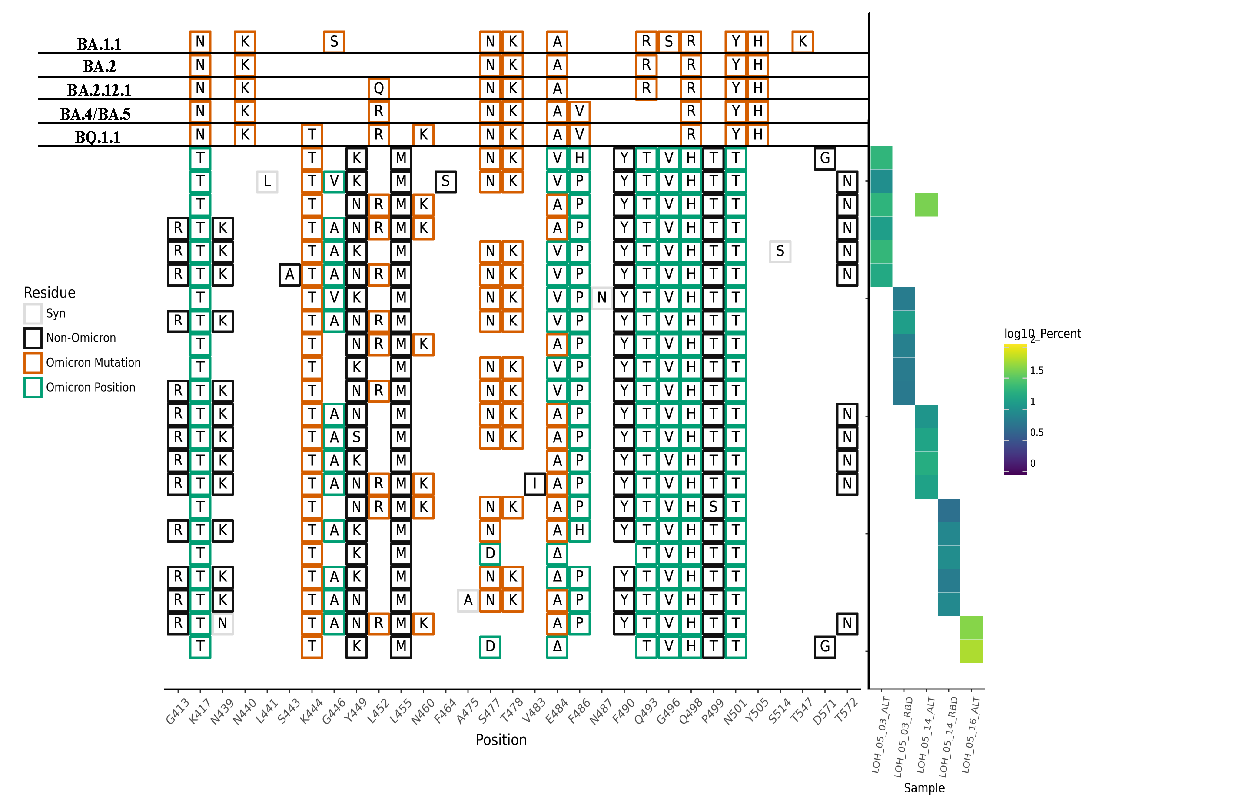


S3 Figure. SARS-CoV-2 haplotype from the second Ohio location.

RBD-focused amplifications of samples collected from the first location in Ohio. Amplifications using the omicron exclusion primer sets are designated as ALT.
